# Value of soluble Urokinase plasminogen activator receptor over age as a biomarker of impaired myocardial relaxation

**DOI:** 10.1186/s12877-017-0668-0

**Published:** 2017-11-28

**Authors:** Angela S. Koh, Bhaarathy Velmurugan, Fei Gao, Ru San Tan, Jia-Ing Wong, Louis L. Y. Teo, Bryan M. H. Keng, Serene J. M. Chua, Jian-Min Yuan, Woon-Puay Koh, Christine Cheung

**Affiliations:** 1 National Heart Centre Singapore, 5 Hospital Drive, Singapore, 169609 Singapore; 2 Duke-NUS Medical School, Singapore, Singapore; 3 Institute of Molecular and Cell Biology, Agency for Science, Technology and Research (A*STAR), Singapore, Singapore; 4 Division of Cancer Control and Population Sciences, University of Pittsburgh Cancer Institute, Pittsburgh, PA USA; 5 Department of Epidemiology, Graduate School of Public Health, University of Pittsburgh, Pittsburgh, PA USA; 6 Saw Swee Hock School of Public Health, National University of Singapore, Singapore, Singapore; 7 Lee Kong Chian School of Medicine, Nanyang Technological University, Singapore, Singapore

**Keywords:** Soluble urokinase plasminogen activator receptor, Biomarker, Cardiac function, Cardiovascular disease, Elderly, Ageing

## Abstract

**Background:**

SuPAR is a biomarker that reflects the level of immune activation. As inflammation plays an important role in the ageing process of the cardiovascular system, we hypothesized that suPAR might be a useful predictive biomarker of the ageing heart.

**Methods:**

We performed conventional and tissue Doppler echocardiography and measured plasma suPAR levels.

**Results:**

We studied community adults (n=120, 37.5% female) (mean age: 70.3±9.3 years) without known cardiovascular disease (CVD). Participants with impaired myocardial relaxation were older (84% vs 59% were aged ≥71 years, *p*=0.002), with more diabetes mellitus (27% vs 11%, *p*=0.034). SuPAR levels were higher among participants with impaired myocardial relaxation (3.9 ng/ml vs 3.0 ng/ml, *p*=0.015). At the univariate level, older age (OR 3.6; 95%CI 1.6, 8.5; *p*=0.003), diabetes mellitus (OR 3.04; 95%CI 1.1, 8.8; *p*=0.04), systolic blood pressure (OR 1.03; 95%CI 1.001, 1.1; *p*=0.041) and suPAR levels ≥3.00ng/ml (OR 3.4; 95%CI 1.16, 7.4; *p*=0.002) were associated with impaired myocardial relaxation. In multivariable regression analysis, only older age (OR 2.8; 95%CI 1.1, 6.7; *p*=0.026) and suPAR (OR 2.7; 95%CI 1.2, 6.1; *p*=0.018) remained independently associated with impaired myocardial relaxation. Receiver operating characteristics (ROC) curve analysis revealed an area under the curve (AUC) value of 0.63 (95% CI 0.54, 0.71) for model that included age alone. Addition of suPAR significantly increased AUC value to 0.70 (95%CI 0.60, 0.79), which was significantly larger than the model with age alone (*p*=0.016).

**Conclusion:**

We demonstrate additional ability of suPAR, over age, to predict impaired myocardial relaxation.

**Trial registration:**

ClinicalTrials.gov Identifier: NCT02791139 (Registered May 31, 2016).

## Background

Age-associated changes in cardiovascular (CV) structure and function and the clinical implications of cardiovascular ageing are well established [1–3]. In spite of this knowledge, clinicians have limited tools to ‘detect’ CV ageing, and hence the ability to focus therapeutic efforts on CV ageing is hampered. Many of the pathophysiological changes of age-associated CV changes cannot be detected in the blood. This is in contrast to traditional CV risk factors such as diabetes mellitus and dyslipidemia that have blood-based diagnostic tools.

A chronic inflammatory state mediated by mechanical and humoral factors, is commonly described in ageing-related cardiovascular disease. These proinflammatory mechanisms drive age-associated arterial remodelling and incomplete myocardial relaxation at the onset of diastole, reducing early left ventricular filling rate [4, 5]. A potential blood-based biomarker reflective of this state of chronic inflammation and which is relatively stable, is soluble urokinase plasminogen activator receptor (suPAR) [6]. SuPAR is emerging as a useful biomarker reflective of increased inflammation particularly in cardiovascular cohorts [7–9]. SuPAR originates from the proteolytic cleavage of the membrane-bound receptor (urokinase plasminogen activator receptor, uPAR) from activated immune and endothelial cells and reflects the level of immune activation [10]. As inflammation plays an important role in the pathogenesis of age-related CVD, we hypothesize that suPAR might be of value as a blood-based biomarker, prior to CVD development. This will have immense value in future trials to target ‘age’ as a risk factor, using suPAR as a blood-based target for such interventions.

In this study, we investigated the value of suPAR as an inflammatory biomarker for ageing-related impairments in myocardial relaxation, among elderly community participants without clinical CVD.

## Methods

The subjects were recruited from the Cardiac Aging Study (CAS), a prospective study initiated in 2014 that examines characteristics and determinants of cardiovascular function in elderly adults. CAS participants were recruited from the prospective, population-based cohort, the Singapore Chinese Health Study (SCHS) [11] and directly from the local community. Briefly, the SCHS recruited 63,257 adults aged 45–74 years and residing in Singapore between 1993 and 1998 for long-term study on association between lifestyle factors and disease risks. This study recruited surviving cohort participants who were re-contacted between 2014 and 2016 for Follow-up III, who did not have self-reported history of physician-diagnosed cardiovascular disease (such as coronary heart disease or stroke).

The study sample consisted of men and women who participated in the baseline CAS 2014 examination who had no self-reported history of physician-diagnosed cardiovascular disease (such as coronary heart disease, atrial fibrillation), stroke or cancer. Written informed consent was obtained from participants upon enrolment. The SingHealth Centralised Institutional Review Board had approved the study protocol.

All participants were examined and interviewed on one study visit by trained study coordinators. Participants completed a standardized questionnaire that included medical history and coronary risk factors. Hypertension was defined by current use of antihypertensive drugs or physician-diagnosed hypertension. Diabetes mellitus was defined by current use of anti-diabetic agents or physician-diagnosed diabetes mellitus. Dyslipidemia was defined by current use of lipid-lowering agents or physician-diagnosed dyslipidemia. Smoking history was defined as ever smokers (former or current smoking) or never smokers. Body mass index was calculated as weight in kilograms divided by the square of height in meters. Sinus rhythm status was ascertained by resting electrocardiogram. Clinical data were obtained on the same day as assessment of echocardiography and serum collection.

Using a ALOKA α10 with a 3.5 MHz probe, echocardiography was performed. Standard echocardiography including 2-D, M-mode, pulse Doppler and tissue Doppler imaging, was performed in the standard parasternal and apical (apical 4-chamber, apical 2-chamber and apical long) views, over three cardiac cycles for each subject. Left ventricular ejection fraction (LVEF), left atrial (LA) volume and LA volume index were obtained. Using Doppler echocardiography, trans-mitral flow E and A wave with the sample volume position at the tip of the mitral valve leaflets from the apical 4-chamber view was recorded. Using sample volume at the septal and lateral annulus from apical four chamber view, we obtained pulsed wave tissue Doppler signals. The frame rate was between 80 and 100 frames per second. Systolic, early diastolic and atrial velocities were recorded and expressed as S′, E’, and A, respectively’. All measurements were measured by the same operator (i.e., JIW) and the measurements were averaged over three cardiac cycles.

Antecubital venous blood samples (20–30 ml) were taken from consenting participants in the morning; fasting was not required before blood collection. After collection, the blood samples were immediately placed on ice for transportation and were processed within 6 h to obtain serum samples, which were subsequently stored at −80 °C.

Plasma levels of soluble uPAR were measured using the Human uPAR Quantikine ELISA kit (R&D Systems, cat no. DUP00), according to the manufacturer’s assay procedures. Briefly, 100 μL of assay diluent was added to each well, followed by 50 μL of plasma samples or protein standards. The plate was sealed and incubated at room temperature for 2 h. Subsequently, the plate was washed 4 times before 200 μL of conjugate was added to each well, then incubated for another 2 h at room temperature. Thereafter, the plate was washed again 4 times. 200 μL of substrate solution was then added and incubated for 30 min, away from light. 50 μL of stop solution was added and read at 450 nm within 30 min in order to halt substrate development. We calculated concentration of suPAR from the equation generated from the standard curve.

In this cohort, the mean E to A ratio was 0.84. We used E to A ratio 0.84 as a cut off to define impaired myocardial relaxation and grouped suPAR using its mean value in the group without impaired myocardial relaxation.

We performed bivariate comparisons between subjects with and without impaired myocardial relaxation with the t test for continuous variables and the chi-square test for categorical variables. Continuous variables are reported as a mean with standard deviation (SD).

We constructed multivariable logistic regression models to assess the association of suPAR with impaired myocardial relaxation. The initial univariate logistic regression model examined the individual association with demographic variables and existing medical conditions. Those associated in the univariate analysis with a *p* < 0.05 were candidate suPAR effect modifiers. Then the potential effect medications of these candidates were assessed individually using multivariable logistic regression.

We further evaluated the discrimination of the models by calculating the area under the receiver-operating characteristic (ROC).

All analyses were performed in STATA 13.

## Results

A total of 120 participants (mean age 70.3 ± 9.3 years; 45 women) were included in this analysis. All completed transthoracic echocardiography and blood sampling on the same day. Baseline clinical characteristics of the study sample are presented in Table 1.

**Table 1:** Baseline clinical characteristics of study participants

		Impaired myocardial function (*n* = 74)	Preserved myocardial function (*n* = 46)	Total (*n* = 120)	*P*-value
Age (years)		73.3 ± 4.0	66.5 ± 13.4	70.3 ± 9.4	0.0001
suPAR		3.9 ± 2.2	3.0 ± 1.4	3.5 ± 2.0	0.015
Female gender		29 (39.2%)	16 (34.8%)	45 (37.5%)	0.63
Body mass index (kg/m^2^)		23.7 (2.9)	23.3 (3.4)	23.5 (3.1)	0.45
Ever smoker		20 (27.0%)	7 (15.2%)	27 (22.5%)	0.13
Hypertension		41 (55.4%)	24 (52.2%)	65 (54.2%)	0.73
Dyslipidemia		41 (55.4%)	22 (47.8%)	63 (52.5%)	0.42
Diabetes mellitus		20 (27.0%)	5 (10.9%)	25 (20.8%)	0.034
Systolic blood pressure		155.4 (39.3)	145.0 (13.5)	151.4 (39.3)	0.088
Diastolic blood pressure		75.9 (10.3)	76.0 (12.2)	75.9 (10.3)	0.96
Pulse	<69	25 (33.8%)	22 (47.8%)	47 (39.2%)	0.13
	≥69	49 (66.2%)	24 (52.2%)	73 (60.8%)	
Interventricular septum thickness at end diastole (IVSd) (cm)		0.9 ± 0.2	0.8 ± 0.1	0.8 ± 0.2	0.012
Interventricular septum thickness at end systole (IVSs) (cm)		1.3 ± 0.3	1.2 ± 0.3	1.3 ± 0.3	0.35
Left ventricular internal diameter end diastole (LVIDd) (cm)		4.5 ± 0.5	4.6 ± 0.5	4.5 ± 0.5	0.14
Left ventricular internal diameter end systole (LVIDs) (cm)		2.5 ± 0.4	2.6 ± 0.4	2.5 ± 0.4	0.027
Left ventricular posterior wall end diastole (LVPWd) (cm)		0.8 ± 0.1	0.7 ± 0.1	0.8 ± 0.1	0.074
Left ventricular posterior wall end systole (LVPWs)(cm)		1.4 ± 0.2	1.4 ± 0.2	1.4 ± 0.2	0.62
Left ventricular outflow tract (LVOT) (cm)		2.1 ± 0.1	2.0 ± 0.2	2.1 ± 0.1	0.49
Aortic diameter (Ao) (cm)		3.2 ± 0.4	3.0 ± 0.5	3.1 ± 0.5	0.058
Left atrium (LA) (cm)		3.7 ± 0.5	3.6 ± 0.6	3.7 ± 0.6	0.17
Left ventricular ejection fraction (LVEF) (%)		75.3 ± 6.7	72.7 ± 7.0	74.3 ± 6.9	0.047
*Left ventricular fractional shortening (LV FS) (%)*		44.3 ± 6.0	42.5 ± 6.3	43.6 ± 6.2	0.13
Left ventricular mass (LVM) (grams)		132.5 ± 41.2	127.6 ± 33.1	130.6 ± 38.2	0.51
Left ventricular mass index (LVMI) (grams/m^2^)		82.0 ± 23.3	78.5 ± 19.2	80.8 ± 21.9	0.43
Left atrial volume (LAV) (ml)		38.0 ± 13.5	36.7 ± 13.6	37.5 ± 13.5	0.62
Left atrial volume index (LAVI) (ml/m^2^)		23.6 ± 7.9	22.3 ± 8.0	23.1 ± 7.9	0.42
*Peak* velocity flow in early diastole E (MV E Peak) (m/s)		0.6 ± 0.1	0.7 ± 0.2	0.7 ± 0.2	0.0001
*Peak* velocity flow in late diastole by atrial contraction A (MV A Peak) (m/s)		0.8 ± 0.2	0.7 ± 0.2	0.8 ± 0.2	0.0014
Ratio of MV E peak/MV A peak (E/A Ratio)		0.8 ± 0.2	1.1 ± 0.4	0.9 ± 0.3	<0.0001
Deceleration time (DT) (m/s)		218.0 ± 39.0	198.0 ± 37.1	210.3 ± 39.4	0.0064
Pulmonary artery systolic pressure (PASP) (mmHg)		26.9 ± 6.1	27.0 ± 6.7	27.0 ± 6.3	0.94
Pulmonary vein systolic velocity (PV-S) (cm/s)		59.2 ± 11.7	59.1 ± 14.7	59.2 ± 11.7	0.96
Pulmonary vein diastolic velocity (PV-D) (cm/s)		46.8 ± 15.0	51.1 ± 14.1	48.5 ± 14.7	0.13
Pulmonary vein flow at atrial contraction (PV-Ar) (ms)		92.8 ± 17.6	95.1 ± 23.4	93.7 ± 20.0	0.55
Mitral inflow duration at atrial contraction (MV A duration) (ms)		115.8 ± 15.6	113.8 ± 17.2	115.0 ± 16.2	0.51
Peak systolic septal mitral annular velocity (Septal S′) (m/s)		0.08 ± 0.01	0.08 ± 0.01	0.08 ± 0.01	0.72
Peak early diastolic septal mitral annular velocity (Septal E’) (m/s)		0.06 ± 0.02	0.09 ± 0.02	0.07 ± 0.02	<0.0001
Septal mitral annular velocity during atrial contraction (Septal A’) (m/s)		0.1 ± 0.02	0.1 ± 0.02	0.1 ± 0.02	0.69
Ratio of MV E peak/Septal E’		10.1 ± 2.7	9.4 ± 4.4	9.8 ± 3.5	0.28

Continuous data are shown as mean ± SD

Participants with impaired myocardial relaxation were older (83.8% vs 58.7% were aged ≥71 years, *p* = 0.002), with more diabetes mellitus (27.0% vs 11.0%, *p* = 0.034). Participants with impaired myocardial relaxation had larger septal wall thickness at end diastole (IVSD) (0.9 ± 0.2 vs 0.8 ± 0.1), *p* = 0.012), smaller left ventricular internal diameter at end systole (LVIDS) (2.5 ± 0.4 vs 2.6 ± 0.4, *p* = 0.027), higher left ventricular ejection fraction (LVEF) (75.3% vs 72.7%, *p* = 0.047), lower peak early phase filling velocity (0.6 ± 0.1 vs 0.7 ± 0.2, *p* = 0.0001), higher peak atrial phase filling velocity (0.8 ± 0.2 vs 0.7 ± 0.2, *p* = 0.0014), lower E/A ratio (0.8 ± 0.2 vs 1.1 ± 0.4, *p* < 0.0001), higher E wave deceleration time (218.0 ± 39.0 vs 198.0 ± 37.1, *p* = 0.0064) and lower peak early diastolic septal mitral annular velocity (0.06 ± 0.02 vs 0.09 ± 0.02, *p* < 0.0001). SuPAR levels were higher among participants with impaired myocardial relaxation (3.9 ± 2.2 ng/ml vs 3.0 ± 1.4 ng/ml, *p* = 0.015).

At the univariate level, older age (OR 3.6, 95%CI 1.6, 8.5; *p* = 0.003), diabetes mellitus (OR 3.04, 95%CI 1.1, 8.8; *p* = 0.040), systolic blood pressure (OR 1.03, 95%CI 1.001, 1.1; *p* = 0.041) and suPAR levels ≥3.00 ng/ml (OR 3.4; 95%CI 1.6, 7.4; *p* = 0.002) were associated with impaired myocardial relaxation. After adjusting for age, diabetes mellitus and systolic blood pressure respectively, suPAR remained independently associated with impaired myocardial relaxation (all OR > 2.5; *p* < 0.05) (Table 2).

**Table 2:** Univariable and multivariable association with impaired myocardial function

		Univariate		Multivariate			
		Unadjusted OR (95% CI)	*P*-value	Adjusted OR (95% CI)	*P*-value	Adjusted OR of suPAR (95% CI)	*P*-value
suPAR	<3.0	1	0.002	–		–	
	≥3.0	3.4 (1.6, 7.4)					
Age (years)	≤70	1	0.003	1			
	>70	3.6 (1.6, 8.5)		2.8 (1.1, 6.7)	0.026	2.7 (1.2, 6.1)	0.016
Gender	Male	1	0.63	–		–	
	Female	1.2 (0.6, 2.6)					
Body mass index (kg/m^2^)		1.05 (0.9, 1.2)	0.44	–		–	
Ever smoker		2.1 (0.8, 5.4)	0.14	–		–	
Hypertension		1.1 (0.5, 2.4)	0.73	–		–	
Dyslipidemia		1.4 (0.6, 2.8)	0.42	–		–	
Diabetes mellitus		3.04 (1.1, 8.8)	0.040	2.4 (0.8, 7.1)	0.12	3.0 (1.4, 6.7)	0.006
Systolic blood pressure		1.03 (1.001, 1.1)	0.041	1.02 (1.0, 1.05)	0.14	3.0 (1.3, 6.5)	0.007
Diastolic blood pressure		1.0 (1.0, 1.03)	0.96	–		–	
Pulse	<69	1	0.13	–		–	
	≥69	1.8 (0.8, 3.8)					

**Fig. 1:**
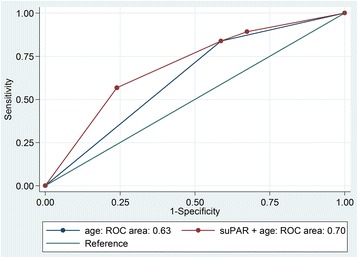
Receiver operating characteristic (ROC) curves for discriminating between preserved myocardial function and impaired myocardial function using age (blue line) or age plus suPAR (red line)

Figure 1 shows that combining suPAR and age increased (*p* = 0.016) the area under the curve (AUC) increased from 0.63 (95% CI 0.54, 0.71) to 0.70 (95% CI 0.60, 0.79).

## Discussion

In this study, we analysed the value of suPAR levels in predicting ageing-related impairments in myocardial relaxation. We provide novel evidence that suPAR adds incremental value over age, in predicting impaired myocardial relaxation that is commonly observed in aged cohorts. This suggests that suPAR is a potential candidate that can be used as a blood-based biomarker of the ageing heart, superior to age.

Our study participants were predominantly old without clinical cardiovascular disease. The impairments in myocardial relaxation were associated with chronological ageing, independent of risk factors. Therefore, our data fills an important gap in geriatric cardiology, where in the lack of blood-based tools to predict risk of ageing-related myocardial dysfunction is hampering clinicians’ ability to identify elderly with early alterations in myocardial function.

In the area of early diastolic dysfunction, hardly any study has looked at the association between suPAR and subclinical myocardial dysfunction. One recent study investigated associations between suPAR and subclinical diastolic dysfunction among a cohort wholly composed of Type 1 diabetic patients [12]. In their study, suPAR was associated with early systolic and diastolic myocardial impairment, measured via echocardiographic techniques [12]. By demonstrating that suPAR is predictive of impaired myocardial relaxation, an early manifestation diastolic dysfunction commonly associated with ageing, our current study builds on this emerging link between suPAR and diastolic function, particularly as assessed by echocardiography. Diabetes mellitus was not a significant predictor of myocardial function in our study, in contrast to Theilade et al.’s study [12] that was prespecified to study suPAR among diabetic patients. Further, we had acquired echocardiographic images simultaneously with blood sampling at the same study visit, possibly eliminating potential clinical events intervening between assessment of myocardial function and blood sampling that may inaccurately depict the relationship between suPAR and myocardial function. In contrast to Theilade’s study [12] that had a median interval of 116 days between echocardiography acquisition and blood sampling, the observed value of suPAR in predicting myocardial function in our study is unlikely influenced by other unknown systemic disease processes. This is especially pertinent in studies that evaluate suPAR as a potential biomarker of disease, because suPAR may represent general states of ill-health. SuPAR predicts a broad spectrum of conditions including cancer, cardiovascular disease, diabetes, kidney disease and mortality [13], purportedly as a result of its role as an endpoint effector of the immune system.

A proinflammatory microenvironment and low-grade inflammation mediated by mechanical and humoral factors, and driven by oxidative stress is commonly described in ageing. These proinflammatory mechanisms drive age-associated arterial remodelling [4], as a result of processes that alter cellular and matrix structure and function. Prolonged contraction of the left ventricle against a stiff aorta results in incomplete myocardial relaxation at the onset of diastole, reducing early left ventricular filling rate. Other structural changes occurring within left ventricular tissue with ageing may also contribute to this reduction in peak LV filling rate [5]. It is possible that the process of inflammation in ageing is mechanistically linked to suPAR release. Proinflammatory cytokines such as tumor necrosis factor-α that induce left ventricular dysfunction may simultaneously stimulate release of suPAR from activated neutrophils, monocytes and endothelial cells via proteolytic cleavage from the cell surface into the plasma [4]. This inflammatory process is maintained by suPAR because it acts as a chemotactic agent that promotes recruitment of immune cells to sites of acute inflammation [10]. The associations between suPAR and subclinical myocardial function in our study provides hypothesis-generating data to suggest a common inflammatory basis for the observed associations [14].

The question as to whether suPAR is a surrogate marker or a contributor to impaired myocardial function associated with the ageing heart requires future work. We believe our study is the first to study suPAR in relation to the ageing heart, particularly in exploring the added ability of suPAR over age in predicting heart ageing.

SuPAR has recently been shown to be associated with hard outcome events in CVD cohorts. A recent study of chronic heart failure patients demonstrated prognostic value of suPAR in predicting all-cause mortality [8]. Another population-based study of elderly participants associated elevated levels of suPAR with increased incidence of incident cardiovascular events [7]. Our results suggest that suPAR may actually be used upstream prior to clinical heart disease, for identifying the ageing heart. Further, future therapeutic efforts that target anti-inflammatory processes, may use suPAR to identify patients for earlier or intensified anti-inflammatory therapies. Treatment effects could also theoretically be monitored through alterations in suPAR levels during treatment [9].

Finally, the relative stability of suPAR in plasma better reflects the state of chronic immune activation compared to other inflammatory biomarkers, such as C-reactive protein (CRP). SuPAR is less inducible than CRP with no major variation in blood and circadian levels [13]. This is particularly relevant for community based studies, such as ageing studies, where blood draws are taken at random times of the day and blood is usually stored prior to analysis necessitating frequent thawing and freezing cycles [6, 13].

The current study has limitations. The analyses are cross-sectional and hence we are unable to determine causality. The sample size is relatively small and participation is voluntary, introducing possible biases in participant selection and inclusion of participants who were generally more interested in their health status (i.e, healthier). Even so, the true relationship between suPAR and myocardial function may only be underestimated, and not overestimated. uPAR has three domains D1, D2 and D3. Soluble forms are composed of D1-D2-D3 and D2-D3 which arise from cleavage of GPI-anchor. Further cleavage of the linker region gives rise to D1 domain [15]. The assay used in our analysis could not differentiate between the forms of suPAR. Hence, we are unable to examine associations of different subtypes of suPAR to myocardial function.

## Conclusions

In this community-based study of participants without cardiovascular disease, we demonstrate additional ability of suPAR, over age, to predict impaired myocardial relaxation. Future studies investigating whether suPAR has a direct role in the pathogenesis of diastolic dysfunction or is merely a bystander reflecting chronic inflammation are warranted.

## Abbreviations

Ao: Aortic diameter
CV: Cardiovascular
DT: Deceleration time
IVSd: Interventricular septum thickness at end diastole
IVSs: Interventricular septum thickness at end systole
LA: Left atrium
LAV: Left atrial volume
LAVI: Left atrial volume index
*LV FS*: *Left ventricular fractional shortening*
LVEF: Left ventricular ejection fraction
LVIDd: Left ventricular internal diameter end diastole
LVIDs: Left ventricular internal diameter end systole
LVM: Left ventricular mass
LVMI: Left ventricular mass index
LVOT: Left ventricular outflow tract
LVPWd: Left ventricular posterior wall end diastole
LVPWs: Left ventricular posterior wall end systole
MV A duration: Mitral inflow duration at atrial contraction
MV A Peak: *Peak* velocity flow in late diastole by atrial contraction A
MV E Peak: *Peak* velocity flow in early diastole E
PASP: Pulmonary artery systolic pressure
PV-Ar: Pulmonary vein flow at atrial contraction
PV-D: Pulmonary vein diastolic velocity
PV-S: Pulmonary vein systolic velocity
Septal A’: Septal mitral annular velocity during atrial contraction
Septal E’: Peak early diastolic septal mitral annular velocity
Septal S′: Peak systolic septal mitral annular velocity
sUPAR: soluble urokinase plasminogen activator receptor
